# Reinforcing Poly(methyl methacrylate) with Bacterial Cellulose Nanofibers Chemically Modified with Methacryolyl Groups

**DOI:** 10.3390/nano12030537

**Published:** 2022-02-04

**Authors:** Hiroyuki Kono, Haruto Tsujisaki, Kenji Tajima

**Affiliations:** 1 Division of Applied Chemistry and Biochemistry, National Institute of Technology, Tomakomai College, Tomakomai 059-1275, Japan; 2 Graduate School of Chemical Sciences and Engineering, Hokkaido University, Sapporo 060-8628, Japan; tsujisaki.haruto.e0@elms.hokudai.ac.jp; 3 Faculty of Engineering, Hokkaido University, Sapporo 060-8628, Japan; ktajima@eng.hokudai.ac.jp

**Keywords:** cellulose nanofiber, nanofibrillated bacterial cellulose, fiber-reinforced resin, poly(methyl methacrylate), silane coupling agent, organosilanes, surface modification, nanofiller

## Abstract

Nanofibrillated bacterial cellulose (NFBC), a type of cellulose nanofiber biosynthesized by *Gluconacetobacter* sp., has extremely long (i.e., high-aspect-ratio) fibers that are expected to be useful as nanofillers for fiber-reinforced composite resins. In this study, we investigated a composite of NFBC and poly(methyl methacrylate) (PMMA), a highly transparent resin, with the aim of improving the mechanical properties of the latter. The abundant hydroxyl groups on the NFBC surface were silylated using 3-(methacryloyloxy)propyltrimethoxysilane (MPTMS), a silane coupling agent bearing a methacryloyl group as the organic functional group. The surface-modified NFBC was homogeneously dispersed in chloroform, mixed with neat PMMA, and converted into PMMA composites using a simple solvent-casting method. The tensile strength and Young’s modulus of the composite increased by factors of 1.6 and 1.8, respectively, when only 0.10 wt% of the surface-modified NFBC was added, without sacrificing the maximum elongation rate. In addition, the composite maintained the high transparency of PMMA, highlighting that the addition of MPTMS-modified NFBC easily reinforce PMMA. Furthermore, interactions involving the organic functional groups of MPTMS were found to be very important for reinforcing PMMA.

## 1. Introduction

Poly(methyl methacrylate) (PMMA) is a widely used transparent thermoplastic material known for its applicability to a variety of products, including car windows and smartphone screens. PMMA has excellent properties; it is easy to shape, is 100% recyclable, and is an excellent alternative to expensive and less-resilient glass [1]. On the other hand, this material sometimes fractures or cracks during use due to mechanical strength issues [1,2,3]. Many approaches for improving the physical properties of PMMA have been explored [4]. A popular way of enhancing the mechanical properties of PMMA involves producing composite materials with microfillers, such as glass [5], polyethylene [6], aramid fiber [7], carbon fiber [8], silica or glass particles [8,9], and stainless steel mesh [10]. According to previous studies [5,6,7,8,9,10], these fillers slightly increase the flexural and impact strengths of PMMA; however, their use is limited because the addition of the filler generally reduces the transparency of the polymer [9], which is one of the most important features of PMMA resin. At the same time, the formation of a PMMA/filler interface often reduces the tension strain limit owing to the effects of structural heterogeneity and interfacial adhesion [11,12,13]. Therefore, various types of micro- and nanofiber, including carbon, aramid, and ultra high-molecular-weight polystyrene, have been investigated as reinforcing fillers that enhance the mechanical properties of PMMA [7,8,14]. Unfortunately, these fillers absorb stress and energy inadequately, which leads to structural deficiencies, a lack of rigidity, and inhomogeneous filler setting that, in turn, result in a fragile and fractured composite [15].

Cellulose nanofiber (CNF) is a potential new filler for PMMA nanocomposites with improved properties because of its large surface area and good stress-transfer features. CNFs are normally obtained from wood pulp by intensive mechanical treatment, including grinding and high-pressure homogenization [16,17,18]. Generally, CNF nanofibers are several micrometers long and 3–100 nm wide; they are also sufficiently thin compared to the wavelength of visible light. Hence, CNFs are expected to usefully reinforce transparent resins, including acrylic and epoxy resins, without scattering visible light and without significant loss of transparency [16,17,18].

Bacterial cellulose (BC), which is biosynthesized by culturing cellulose-producing bacteria of the *Gluconacetobacter* sp. genus in a medium containing D-glucose under static conditions, forms CNFs that are different to pulp-derived CNFs. In general, BC is obtained as a gel-like matrix of ~100 nm-wide cellulose fibrils that are three-dimensionally intertwined and contain large amounts of water [19,20,21]. The use of the swirling culture method during BC synthesis has recently been reported to inhibit fiber formation due to the generation of torque, resulting in a water-dispersed form of BC [22,23,24]. The obtained cellulose fibers are approximately 20 nm wide, which is approximately one-fifth that of the BC obtained by static culturing. This water-dispersible BC is referred to as nanofibrillated bacterial nanocellulose (NFBC). The formation of fibers that are more than 15 μm long with extremely high aspect ratios is one advantage of NFBC [22], which can possibly be used as a fiber-reinforcing filler for resins, including PMMA [13,25].

The surface layers of NFBC and CNF contain large numbers of hydroxyl groups that need to be considered when using these cellulosic nanofibers as fiber-reinforcing fillers for resins; consequently, these surface hydroxyl groups need to be substituted with functional groups that have high affinities for the resin to facilitate adhesion [18,25,26,27]. However, nanofibers tend to aggregate in the organic solvent in which most substitution reactions involving cellulose are performed. In addition, hydrogen bonds form between nanofiber surfaces as the cellulosic nanofibers are dried, resulting in inferior features. Therefore, a silane-coupling reaction that proceeds in aqueous media is considered the most effective method for modifying the surfaces of cellulosic nanofibers. A method for substituting the surface hydroxyl groups of NFBC with aminopropylsilyl groups through silane coupling with 3-aminopropyltrimethoxysilane (APTMS) was recently reported [18]. Compositing PMMA with NFBC modified by APTMS led to superior tensile strengths while maintaining the transparency of PMMA. On the other hand, the addition of the modified NFBC led to lower tension strain limits and PMMA with inferior impact strengths.

**Scheme 1:**
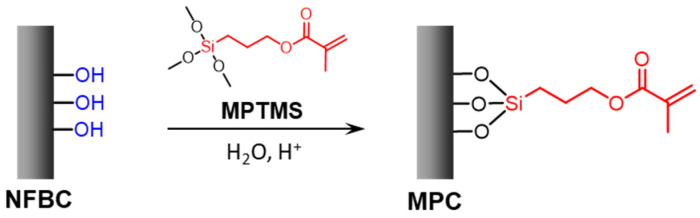
Scheme showing how the nanofibrillated bacterial nanocellulose (NFBC) surface is modified with 3-(methacryloyloxy)propyltrimethoxysilane (MPTMS). Surface hydroxyl groups are silylated with MPTMS in aqueous solution to form 3-(methacryloyloxy)propyltrimethoxysilylated nanocellulose (MPC).

In this study, the surface layers of NFBCs were treated with 3-(methacryloyloxy)propyltrimethoxysilane (MPTMS), which is a silane coupling agent bearing methacryloyl groups that are structurally similar to the side chains of PMMA (Scheme 1). This treatment protocol is designed to enhance PMMA/NFBC interfacial compatibility by improving structural similarity. PMMA composites containing the MPTMS-modified NFBCs were prepared with the aim of improving the tensile mechanical properties of PMMA, including its tensile stress limit, Young’s modulus, and tension strain limit. The effect of the modified-NFBC concentration in the PMMA composite was evaluated in terms of the transparency and tensile mechanical properties of the product, which confirmed that NFBC is a superior fiber-reinforced nanofiller. In addition, molecular-level interactions between the methacryloyl groups on the modified NFBC and the PMMA resin are discussed by comparing the results obtained in this study with previously reported fiber-reinforcement data for APTMS-modified NFBC [18].

## 2. Materials and Methods

### 2.1. Materials

The 1.0% (*w/v*) NFBC suspension in water was prepared according to a previously reported method [23,24] and kindly provided by Kusano Sakko Inc., Ebetsu, Japan. The precise concentration of the NFBC suspension is based on the dry weight determined for a 10 mL NFBC suspension using an MS-70 infrared moisture meter (A&D Co., Tokyo, Japan). MPTMS was purchased from Tokyo Chemistry Industry Co. (Tokyo, Japan). PMMA (weight-average molecular weight: 120 kDa) was purchased from Sigma-Aldrich, St. Louis, Missouri, USA. Other chemicals were very pure (analytical grade) and all solutions were prepared using deionized water.

### 2.2. Modifying the Nanofibrillated Bacterial Nanocellulose (NFBC) Surface: Preparing 3-(Methacryloyloxy)Propyltrimethoxysilylated Nanocellulose (MPC)

The 1.0% (*w/v*) NFBC suspension (300 mL, dry weight: 3 g; 19 mmol of anhydroglucose units (AGU)) was concentrated by centrifugation at 9000 g for 20 min, after which the pellet was suspended in water to a concentration of 2.0% (*w/v*). The pH of the suspension was adjusted to 4 using 0.5 mol L^−1^ HCl solution, after which 4.4 mL (19 mmol) of MPTMS was added dropwise to the suspension over 15 min. The mixture was vigorously stirred at 700 rpm at 30 °C for 4 h using a Teflon impeller, after which the product was collected by centrifugation at 10,000 g for 20 min, washed three times with acetone and water, and then freeze-dried to obtain MPC 1. MPC 2 and MPC 3 were similarly prepared by changing the amount of MPTMS to 13.2 mL (56 mmol) and 39.6 mL (167 mmol), respectively.

### 2.3. Structures and Morphologies of the MPC Samples

#### 2.3.1. Fourier-Transform Infrared (FTIR) Spectroscopy

FTIR spectra of NFBC and MPC 1–3 were recorded on a Spectrum II FTIR spectrometer (PerkinElmer, Inc., Waltham, MA, USA) at a resolution of 1 cm^−1^ in the 4100–600 cm^−1^ range and averaged over 16 scans.

#### 2.3.2. Solid-State ^13^C Nuclear Magnetic Resonance (NMR) Spectroscopy

Solid-state ^13^C NMR spectra were acquired at 298 K on a Bruker AVIII500 spectrometer equipped with a 4 mm magic angle spinning (MAS) probe (Bruker BioSpin GmbH, Rheinstetten, Germany). The dipolar-decoupled MAS ^13^C NMR spectra of NFBC and MPC 1–3 were recorded by setting the MAS frequency, ^13^C excitation pulse length (flip angle of 30°), data acquisition time, and repetition time to 10 kHz, 1.35 μs, 15, and 30 s, respectively. Chemical shifts were calibrated against the carbonyl signal (176.03 ppm) of an external D-glycine standard.

#### 2.3.3. X-ray Diffractometry (XRD)

XRD patterns of NFBC and MPC 1–3 were recorded at 298 K on a Bruker D8 Advance diffractometer (Bruker AXS GmbH, Karlsruhe, Germany) using Ni-filtered CuKα (λ = 1.54056 Å) radiation generated at 40 kV and 40 mA, and at scan rate of 1.000°/min in the 5–35° 2*θ* range. The crystallinity indices (CIs) of these samples were calculated from the areas of their crystalline cellulose peaks relative to the total areas of their crystalline + amorphous cellulose peaks [18,25,28]. TOPAS software (Ver. 4.2, Bruker AXS GmbH, Karlsruhe, Germany) was used to fit the X-ray diffractograms by using a non-linear least-squares fitting [18].

#### 2.3.4. Thermal Gravimetry (TG)/Differential Thermal Analysis (DTA) 

TG/DTA traces were acquired on an EVO2 TG 8120 Plus thermogravimetric dynamic thermal analyzer (Rigaku, Akishima, Japan) using an Al_2_O_3_ crucible under a flow of nitrogen gas. Each sample weighed 7 mg. Thermograms were obtained in the 50–500 °C range at 5 °C min^−1^.

#### 2.3.5. Microscopy

Morphologies were examined by field-emission scanning electron microscopy (SEM; JSM-7500F, JEOL Ltd., Akishima, Japan). A 50 μL aliquot of each 0.10 wt% MPC suspension in chloroform, or NFBC suspension in water, was dropped onto an aluminum SEM grid. The solvent had evaporated at 40 °C for 30 min, after which the specimen was coated with a 4 nm-thick platinum layer and then observed at an accelerator voltage of 5 kV. *ImageJ* software (Ver. 1.53j13, Wayne Rasband, National Institutes of Health, Kensington, MD, USA) was used to determine the width distribution of each sample, with each average width calculated using 100 randomly selected points on nanofibers in the corresponding SEM image.

### 2.4. Transparency Testing

NFBC and MPC dispersions in water or chloroform (1 wt%, 10 mL) were homogenized using a T25 digital Ultra-Turrax disperser (IKA-Werke GmbH, Staufen im Breisgau, Germany) equipped with an 8G-ST stator shaft (diameter: 8 mm) at 15,000 rpm for 5 min. After standing for 2 h, the transparency of the suspension was measured in the 340–750 nm wavelength range on an Evolution 201 UV-vis spectrometer (Thermo Fisher Scientific Inc., Waltham, MA, USA).

### 2.5. Preparing Poly(Methyl Methacrylate) (PMMA)/MPC Composites and Measuring Their Transparencies 

PMMA (2.0 g) was dissolved in 20 mL of chloroform, after which 1.0, 2.0, 5.0, 10, 20, or 40 mg of MPC 1 was added and completely dispersed using a Polytron P10-35G homogenizer (Kinematica AG, Malters, Switzerland) equipped with a PT-DA 12/2MEC-F154 generator shaft (diameter: 12 mm) at 30,000 rpm for 5 min. Each suspension (10 mL) was cast onto a Teflon Petri dish (diameter: 6.0 cm), after which the solvent was evaporated at 25 °C for 24 h. The sheet was hot-pressed at 160 °C for 30 s with a clearance of 0.5 mm using an H300-10D heat press apparatus (Asone Co., Osaka, Japan) to produce a PMMA/MPC 1 composite film. Film transparencies were evaluated in the 340–750 nm wavelength range using the aforementioned UV-vis spectrometer.

### 2.6. Tensile Mechanical Testing

Dumbbell test specimens (50 mm × 8.5 mm) were prepared by punching cast films with a press-punching machine (Kobunshi Keiki Co., Kyoto, Japan). An AG-X plus 10kNX (Shimadzu Corp., Kyoto, Japan) load frame equipped with a 500 N load cell was used during uniaxial tensile testing. The dumbbell test specimens were subjected to tensile loading at a crosshead speed of 3.0 mm/min.

## 3. Results and Discussion

### 3.1. Characterizing the MPC Structures

#### 3.1.1. FTIR Spectroscopy

**Figure 1:**
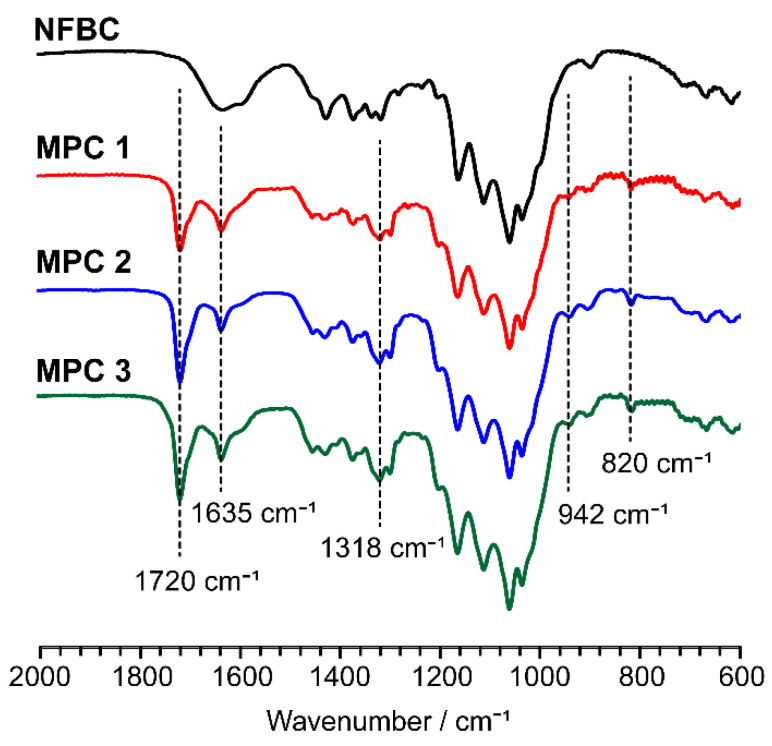
Fourier transform infrared (FTIR) spectra of MPC 1–3 and NFBC.

Figure 1 shows FTIR spectra of MPC 1–3 and NFBC as starting materials. Compared to the FTIR spectrum of NFBC, the spectra of the MPC samples exhibit additional adsorption bands at 1720, 1635, 1318, 942, and 820 cm^−1^ that correspond to C=O stretching, C=C stretching, Si–C bending, C=C out-of-plane bending, and Si–O stretching vibrations, respectively [28,29]. These adsorption bands in the FTIR spectra of the MPC samples reveal that the silane coupling reaction between NFBC and MPTMS proceeded in aqueous solution; the three methoxy groups of MPTMS were solvated by the acid catalyst, and the formed silanol groups reacted with the hydroxyl groups of cellulose to modify the NFBC surface. In addition, the intensities of the new absorption bands were observed to gradually increase as the amount of MPTMS used to prepare the MPC samples was increased, which indicates that the degree to which the NFBC surface is chemically modified depends on the MPTMS concentration in the reaction mixture.

#### 3.1.2. Solid-State ^13^C Nuclear Magnetic Resonance (NMR) Spectroscopy

**Figure 2:**
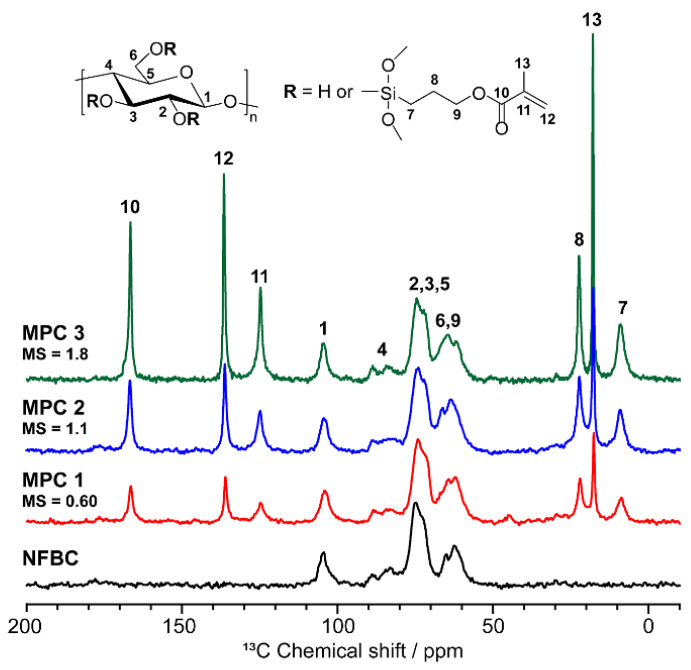
Solid-state ^13^C NMR spectra of MPC 1–3 and NFBC.

**Figure 3:**
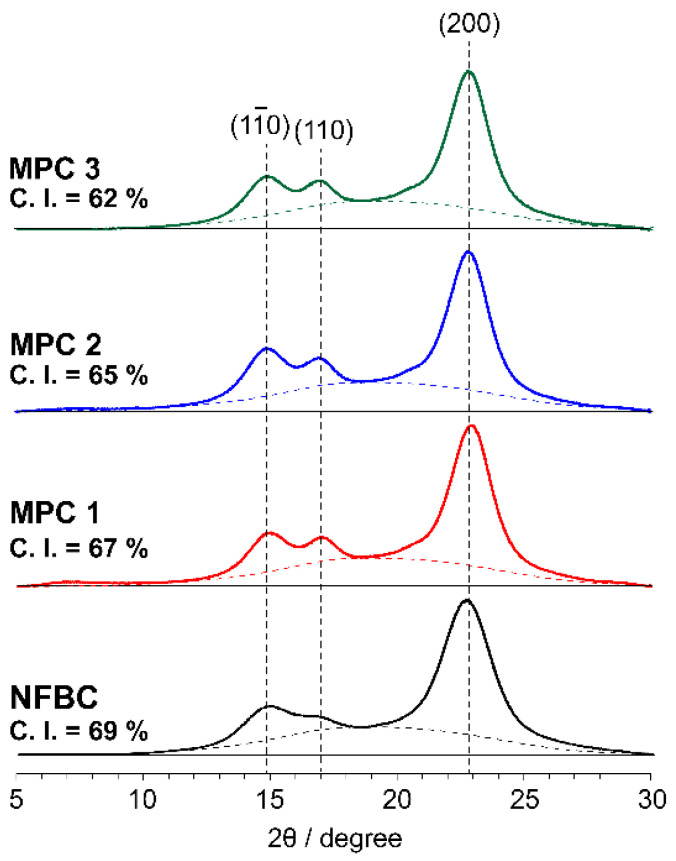
X-ray diffraction (XRD) patterns of MPC 1–3 and NFBC.

Figure 2 shows solid-state ^13^C NMR spectra of MPC 1–3 and NFBC along with resonance assignments. In addition to the six resonances for the AGU of cellulose (carbons 1–6 in Figure 3) [30], the ^13^C NMR spectra of the MPC samples show six resonances at 166, 136, 124, 22, 18, and 9 ppm that are assigned to carbons 10, 12, 11, 8, 13, and 7, respectively, of the 3-(methacryloyloxy)propyl groups [25,28,31]. The ^13^C resonance of remaining carbon 9 overlaps with that of carbon 6 of cellulose at 63 ppm. The degrees of 3-(methacryloyloxy)propylsilyl molar substitution in the MPC samples, which correspond to the average numbers of MPTMS reacted per cellulose AGU, were determined by inserting the sums of the integral values of ^13^C resonances 7–13 (but not 9) relative to those of carbon 1 into the following Equation (1):

(1) $$\mathrm{MS}=\frac{\left(\mathrm{sum}\;\mathrm{of}\;\mathrm{integral}\;\mathrm{values}\;\mathrm{of}\;\mathrm{resonances}\;7,\;8,\;10\text{–}13\right)}{6}$$

The MS values of MPC 1–3 were determined to be 0.60, 1.1, and 1.8, respectively, which indicate that MPC MS increases with increasing MPTMS used during MPC synthesis.

#### 3.1.3. X-ray Diffraction (XRD)

**Figure 4:**
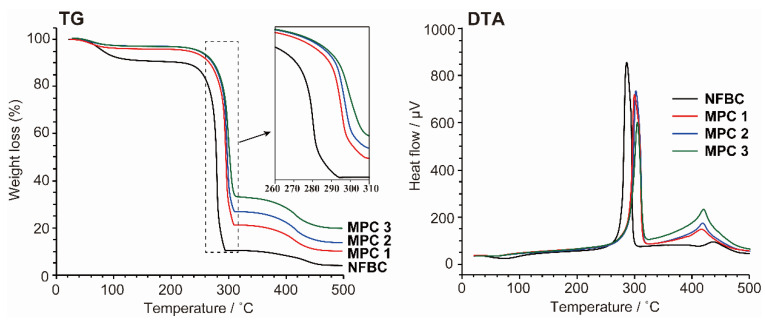
Thermal gravimetry/differential thermal analysis (TG/DTA) data for MPC 1–3 and NFBC.

Figure 3 shows X-ray diffractograms of MPC 1–3 and the NFBC starting material. All samples show characteristic crystalline cellulose I patterns with diffraction peaks at *2θ* = 14.4°, 16.9°, and 22.8° that correspond to the ($1\bar{1}0$), (110), and (200) crystal planes of cellulose I, respectively [25]. Diffractogram curve-fitting enabled the amorphous region of each sample (which corresponds to the area below each dotted line in Figure 4) to be determined. The amorphous areas of NFBC and MPC 1–3 were calculated to be 31%, 33%, 35%, and 38% of the total diffracted area, respectively, providing CI values of 69%, 67%, 65%, and 62%, respectively. No differences in the positions and half-widths of the crystalline cellulose I peaks of the NFBC and MPC samples were observed, suggesting that the inner cellulose structures of MPC 1–3 are the same following silane coupling, and that the reaction proceeds on the surfaces of the CNFs. In addition, since the CIs of MPC 1–3 tend to decrease with increasing sample MS, we conclude that the formed polysiloxane domains on the nanofiber surfaces are structurally amorphous. Hence, the CIs of the MPC samples decrease somewhat with increasing MS.

#### 3.1.4. Thermal Properties

Figure 4 shows TG/DTA data for MPC 1–3 and NFBC. As previously reported, NFBC thermally decomposes through two weight-loss stages: the first (70–140 °C) involves the vaporization of adsorbed water from cellulose chains, while the second (270–290 °C) involves the pyrolytic dehydration and decomposition of cellulose molecules [13], and exhibits a clear endothermic DTA peak at 280 °C. Although MPC 1–3 exhibit similar thermal behavior to that of NFBC, they lose much less weight at 70–140 °C than NFBC, which is consistent with MPC-surface hydrophobization resulting from silane coupling leading to fewer interactions between water molecules and the surface. In addition, the MPC samples exhibit second weight losses due to cellulose pyrolysis that are shifted to higher temperatures than that of NFBC. The decomposition temperature appears to increase with increasing MS, as confirmed by a shift in the endothermic DTA peak to higher temperatures. Moreover, the TG traces of the MPC samples show clear weight losses between 390 and 440 °C, with DTA endothermic peaks detected at 419 °C, which is believed to correspond to the decomposition of the stable siloxane structure formed on the NFBC surface. Based on these thermal analysis data, we conclude that the silane layer on the NFBC surface blocks thermal conduction and increases the pyrolysis temperature of cellulose [13].

### 3.2. MPC Sample Morphologies 

**Figure 5:**
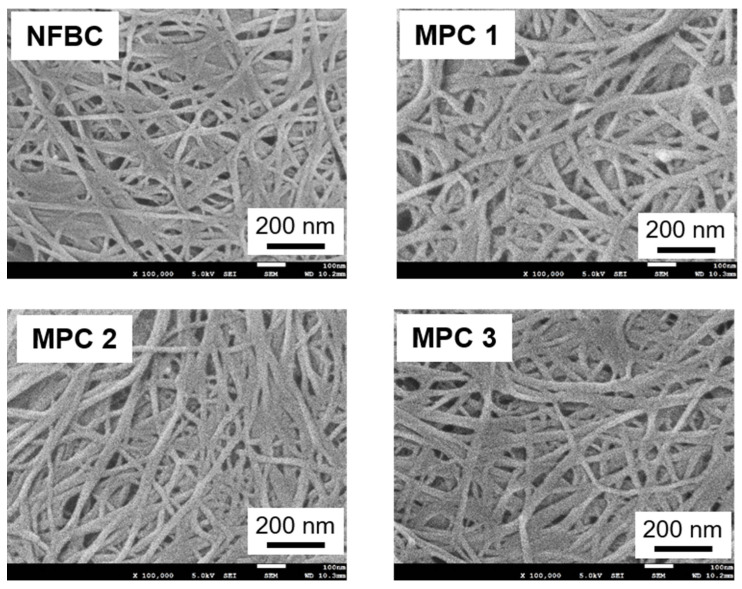
SEM images of NFBC and MPC 1–3.

**Figure 6:**
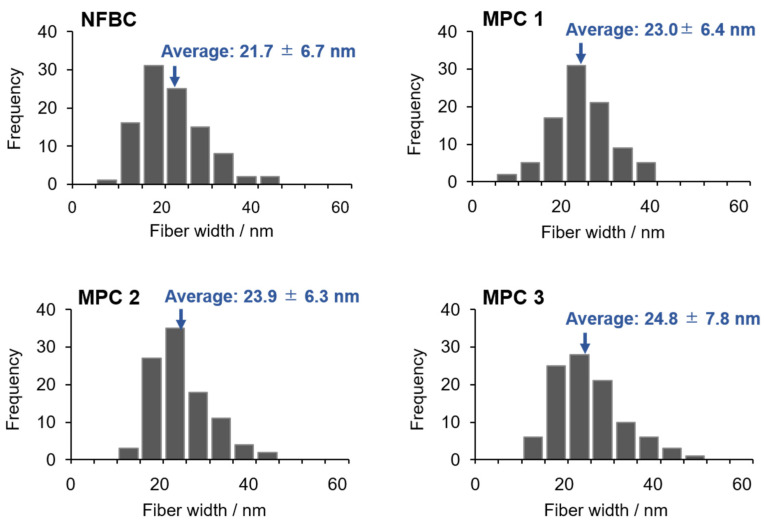
Fiber-width distributions (*n* = 100) of NFBC and MPC 1–3 determined from the scanning electron microscopy (SEM) images in Figure 5.

Figure 5 shows SEM images of NFBC and MPC 1–3. Fine fibrillar structures were observed after silane coupling with MPTMS. The MPC 1–3 fibers are 23.0 ± 6.4, 23.9 ± 6.3, and 24.8 ± 7.8 nm wide, respectively, while the NFBC fibers are 21.7 ± 6.7 nm wide (Figure 6). Cellulose fibers have previously been reported to widen through silane coupling [25]; MPTMS treatment also resulted in MPC fiber diameters that slightly increased with increase MS. These results are consistent with the formation of polysiloxane coatings on the surfaces of the NFBC samples, as suggested by the thermal-analysis data.

### 3.3. Dispersion States of the Silane-Modified NFBC Samples in Water and Chloroform

**Figure 7:**
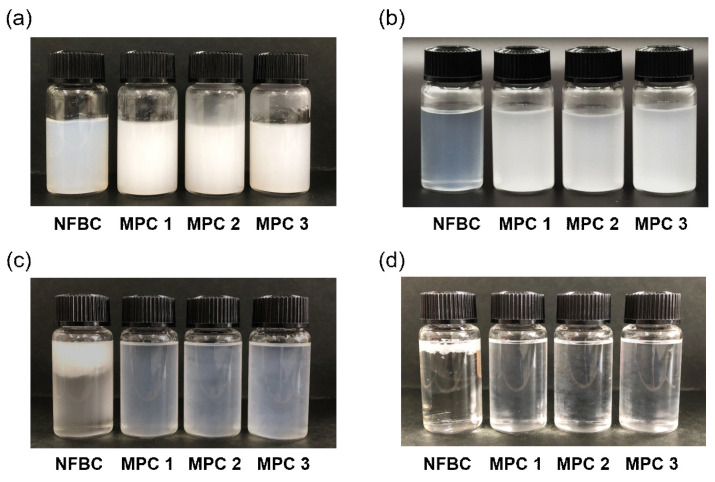
Dispersion states of MPC 1–3 and NFBC: (**a**) 1.0% *w/v* in water, (**b**) 0.1% *w/v* in water, (**c**) 1.0% *w/v* in chloroform, and (**d**) 0.1% *w/v* in chloroform.

Figure 7a,b show the dispersion states of 1.0% *w/v* MPC 1–3 and NFBC in water and chloroform. NFBC was well-dispersed in water but immediately aggregated in chloroform and clumped together in the upper layer due to the hydroxyl groups on its surface. In contrast, MPC samples precipitated and became suspended in water but dispersed well in chloroform. NFBC aggregated in chloroform even when its concentration was reduced to 0.1% *w/v* (Figure 7c), while the MPC samples showed good dispersibilities.

**Figure 8:**
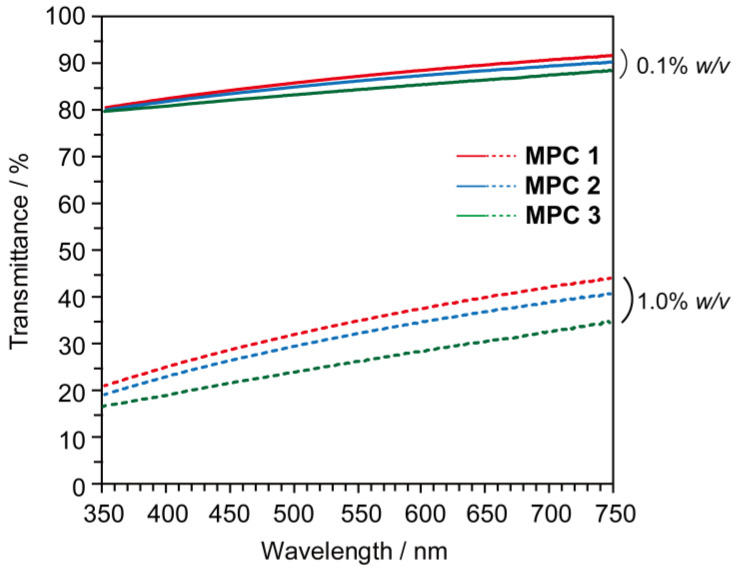
Transparencies of 0.1% *w/v* (solid lines) and 1.0% *w/v* (dotted lines) MPC 1–3 dispersed in chloroform.

Visible-light transmittance data for MPC 1–3 dispersed in chloroform at 1.0 and 0.1% *w/v* are shown in Figure 8; transmittances of 17–42% and 80–89% were observed at the two concentrations, respectively. In addition, the samples tended to be less transmissive at lower wavelengths. These observations reveal that MPC suspensions scatter light; however, because the MPC fibers are less than 50 nm wide (Figure 6), light scattering is likely to be the result of MPC-fiber entanglement that is ascribable to their lengths (>15 μm) [22]. This conclusion is also supported by the observation that water-dispersed NFBCs are not transparent (Figure 7a,b). When the data for the 1.0% *w/v* MPC 1–3 dispersions are compared, transmittance was also observed to decrease with increasing MS, which is believed to be due to fiber diameter, which was observed to increase slightly with increasing MS (Figure 6). On the other hand, the 0.1% *w/v* MPC 1–3 dispersions exhibited almost identical transmittances across the entire visible-light region, although transmittance decreased slightly with increasing MS. These results indicate that all samples with MS values greater than 0.60 show identical interactions between the MPC surface and chloroform, and these MPC samples disperse well in chloroform.

### 3.4. Composites with PMMA

**Figure 9:**
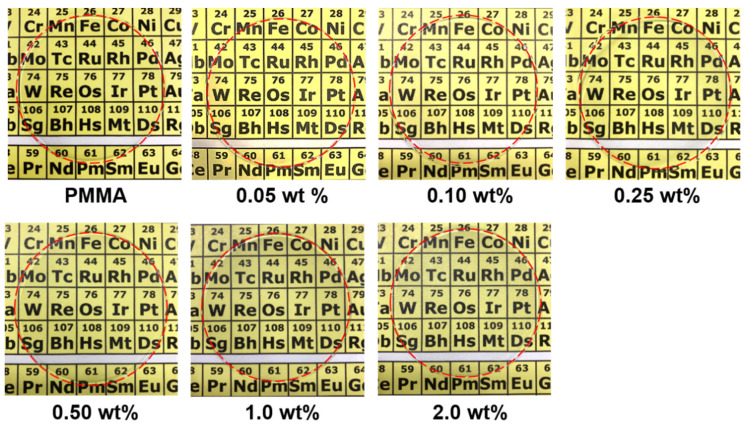
Photographic images of ~0.5-mm-thick PMMA composite sheets containing 0.05, 0.10, 0.25, 0.50, 1.0, and 2.0 wt% MPC 1, with neat PMMA as a reference, overlaid on the periodic table. The perimeter of each sheet is indicated by a red circle.

**Figure 10:**
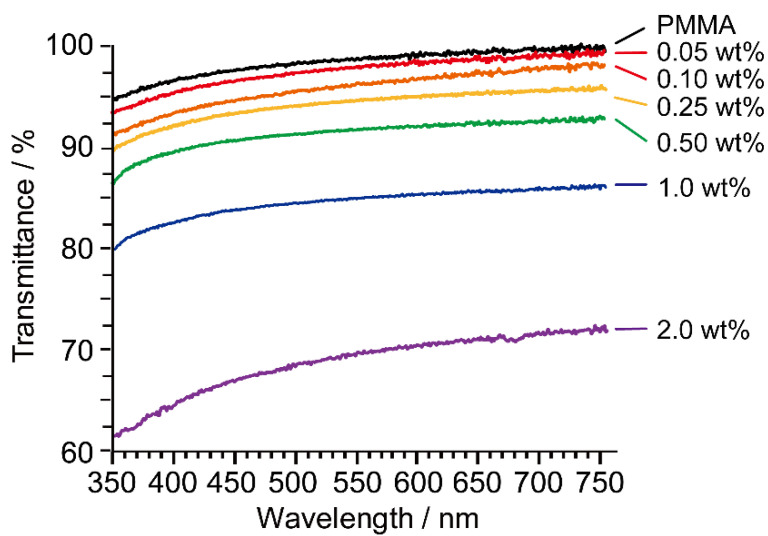
Transmittance spectra of ~0.5-mm-thick PMMA composite sheets containing 0.05, 0.10, 0.25, 0.50, 1.0, and 2.0 wt% MPC 1, with neat PMMA as a reference.

PMMA composites were prepared with MPC 1 as the filler because it exhibited the highest dispersibility in chloroform among the MPC samples prepared in this study (Figure 8). As shown in Figure 9, PMMA composite films blended with 0.05–2.0 wt% of MPC 1 were prepared by casting from chloroform solutions. No fiber agglomeration was visually observed in any of the prepared composite films; however, films containing 1.0% or more MPC were slightly cloudy in appearance. The visible-light (350–750 nm) transmittances of these composites and neat PMMA are shown in Figure 10. Compared to PMMA, the transmittance of the composite was observed to decrease with increasing MPC concentration in the resin. MPC concentrations of 0.50 wt% or less resulted in composite transparencies that were within 10% of that of PMMA, while 1.0 wt% or more interfered with the transparency of PMMA. In other words, a low MPC concentration relative to the resin (i.e., 0.50 wt% or less) is desirable for maintaining the transparency of the PMMA resin when composited with MPC.

### 3.5. Mechanical Properties of the PMMA Composites

**Figure 11:**
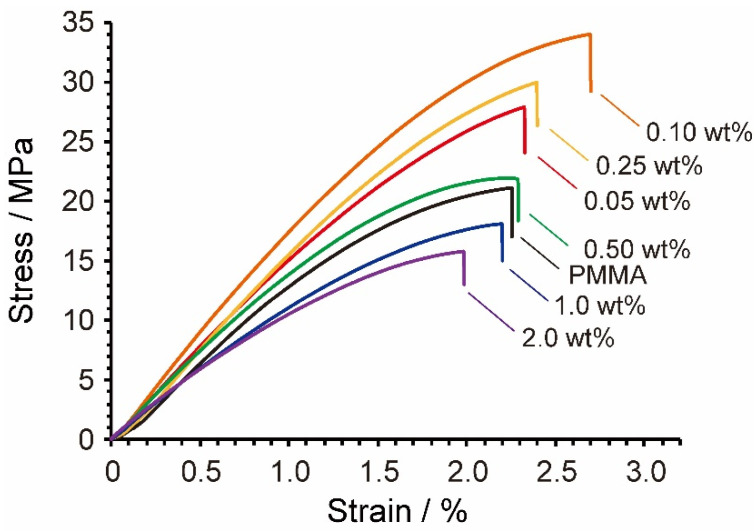
Tensile stress–strain curves for PMMA composites containing 0.05, 0.10, 0.25, 0.50, 1.0, and 2.0 wt% MPC 1, with that for PMMA provided as a reference.

**Figure 12:**
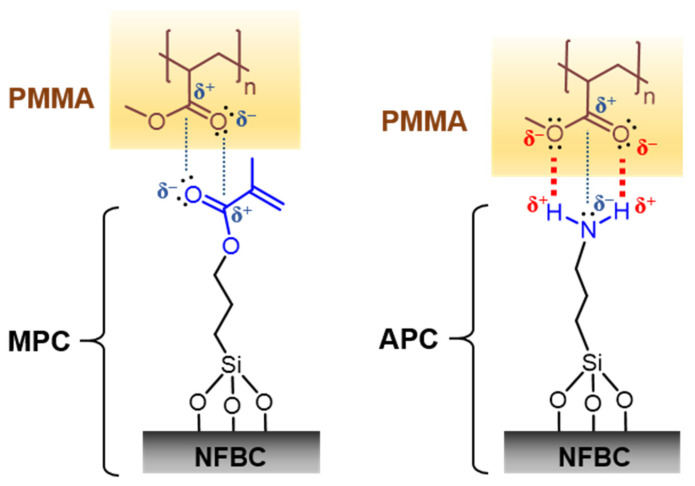
Differences in the molecular structures of NFBCs modified with MPTMS (**left**, MPC) and APTMS (APC, **right**), and intermolecular forces formed with PMMA.

The mechanical properties of dumbbell-shaped specimens of PMMA composite films containing 0.05–2.0 wt% MPC 1 were investigated by tensile stain–stress testing (Figure 11), with neat PMMA film as a reference. Representative tensile strain-stress curves for these composites recorded at a uniform tensile-force rate are shown in Figure 12, with tensile properties summarized in Table 1. Tensile mechanical properties, including the tensile stress limits and Young’s moduli of PMMA composites with MPC 1 concentrations below 0.50 wt% are higher than those of PMMA. Both the tensile stress limit and Young’s modulus increased with increasing MPC 1 concentration, with maxima observed at an MPC concentration of 0.10 wt%. The tensile strength and Young’s modulus of the composite containing 0.1 wt% MPC 1 were determined to be 56% and 82% higher than those of neat PMMA, respectively. Further increases in MPC 1 concentration led to composites with inferior tensile mechanical properties. As was observed for tensile strength, tensile elongation was also observed to increase with increasing MPC 1 concentration to a value of 0.10 wt%, and decreased with further increases in concentration. A maximum tensile elongation of 2.8% was observed for the composite containing 0.10 wt% MPC, while that of neat PMMA is 2.3%. Therefore, the addition of MPC 1 clearly improves the Young’s modulus and tensile stress limit of PMMA without decreasing its tensile elongation.

### 3.6. Interaction between the MPC Surface and PMMA at the Molecular Level

In this study, MPC samples with MS values of 0.60 or higher were found to have sufficiently modified surface layers because they all disperse well in chloroform (Figure 8). In addition, the tensile mechanical properties of PMMA were dramatically enhanced by compositing MPC 1 with PMMA, without any significant loss of transparency (Table 1). Only 0.10 wt% MPC 1 is required to maximize the strength of the PMMA composite; therefore, we conclude that MPC is uniformly dispersed in the PMMA, forming a network structure that improves the mechanical properties of the PMMA composite. On the other hand, inferior mechanical properties and transparencies were observed when 1.0 wt% or more MPC 1 was combined with PMMA, which we conclude to be due to significant differences in the physical properties of MPC and PMMA, such as density, molecular orientation, crystal structure, and crystallinity, which become more pronounced in composites with high MPC 1 concentrations. As a result, the addition of excess MPC resulted in a decrease in the strength of the PMMA composite, as structural heterogeneity with PMMA counteracts the increase in strength associated with the network structure of MPC [32,33,34].

A previous study reported that PMMA is reinforced by NFBC whose surface was chemically modified with APTMS (APC) [13]. The maximum tensile stress limit and Young’s modulus of this composite were reported to be 105% and 155% higher than those of PMMA, respectively, at an optimal APC concentration of 1.0 wt%. However, the addition of APC to PMMA resulted in a considerable decrease in the tensile elongation of the composite, regardless of the APC concentration. On the other hand, the maximum tensile stress limit and Young’s modulus of the PMMA composite reinforced with MPC are 58% and 82% higher than those of PMMA, respectively, which are lower than those reported for the optimal APC/PMMA composite; however, the use of MPC was found to increase the tensile stress limit and Young’s modulus without lowering tensile elongation, which is an advantage of MPC over APC [15]. The differences in the abilities of APC and MPC to reinforce PMMA is likely to be due to differences in the organic functional groups on their surfaces (Figure 12). In the case of the former, amino groups bonded to the NFBC surface through the silane linkage can form up to two hydrogen bonds and one dipole–dipole interaction with the acrylic groups of PMMA to provide strong NFBC/PMMA adhesion [35,36]. On the other hand, the methacryloyl groups of MPC can only form up to two dipole–dipole interactions with the acrylic groups of PMMA [36,37]; hence, the MPC surface adheres less to PMMA than APC. However, the structural similarity between the methacryloyl group of MPC and the PMMA molecule itself increases affinity at the MPC/PMMA interface, leading to a maximum tensile strength at a concentration of only 0.1 wt% without any decline in the tensile elongation of the composite.

### 3.7. Applying NFBCs Surface-Modified with Organosilanes

The silane coupling reaction can be conducted in an aqueous solution, which is an advantage of the MPTMS-modified NFBC. Drying the original suspension prior to surface modification is important, since most CNFs are generally dispersed in water. Hydrogen bonds are generally formed between nanofibers when CNFs are dried, which diminishes the favorable properties of the CNFs. Therefore, the process used to chemically modify the NFBCs with the silane coupling agents used in this study can be applied to CNF suspensions in water, with the expectation that their surface properties can easily be modified without fiber aggregation. Silane coupling agents bearing a variety of functional groups are currently available, which facilitates CNF and NFBC surface modification for various engineering, medical, and pharmaceutical applications [38,39,40].

### 3.8. Silicon Atom States on the MPC Surface

**Figure 13:**
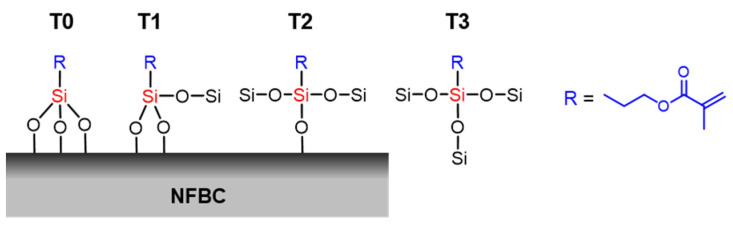
Schematic showing monomeric (T0), singly (T1), doubly (T2), and fully (T3) condensed silicon atoms on the NFBC surface.

MPTMS contains three hydrolyzable methoxy groups. Surface modification begins with the solvolysis of these methoxy groups by an acid catalyst prior to any reaction with the cellulose surface. The formed silanol groups then react with the hydroxyl groups of cellulose to modify the cellulose surface. Consequently, silicon atoms can couple to cellulose in four possible ways: monomerically, or through singly, doubly, or fully condensed silicon atoms, which are referred to as T0, T1, T2, and T3, respectively (Figure 13). These four silicon-atom states are distinguishable by solid-state ^29^Si NMR spectroscopy [41]. In our previous study, in which we structurally characterized APC samples using this NMR technique, T0 was found to exist negligibly when the MS value of the APTMS of the APC exceeded 0.27, with most silicon atoms existing in T1, T2, and T3 states that form polysiloxane phases on the NFBC surface [13]. In this study, the MS values of MPC 1–3 were determined to be 0.60, 1.1, and 1.8, respectively (Figure 1), which suggests that most MPTMS molecules bound to NFBC condense with each other to form polysiloxanes. The formation of polysiloxanes on the NFBC surface is supported by higher average MPC fiber diameters (Figure 6) and MPC dispersions transmittances in chloroform that slightly decrease with increasing MS (Figure 8). Moreover, the pyrolysis temperature of the cellulose domain in the MPC sample was also observed to shift to higher temperature with increasing MS (Figure 4), which also supports the conclusion that the NFBC surface is coated by polysiloxanes.

Therefore, a di- or monoethoxysilane can be used instead of a triethoxysilane (such as MPTMS) to minimize polysiloxane formation while ensuring a uniformly modified NFBC surface [42]. The use of these silane coupling agents is expected to result in a smaller increase in NFBC fiber diameter and improved interactions with the resin; it may also contribute to reducing the amount of silane coupling agent used and to improving the transparency of the composite. We will modify the surface layers of the composites with various silane coupling agents and statistically evaluate their bonding states and physical properties in future studies.

## 4. Conclusions

In this study, we found that NFBC surface-treated with MPTMS bearing methacryloyl groups enhances the tensile mechanical properties of PMMA at a concentration of only 0.10 wt% while maintaining the transparency of PMMA. In addition, we found that the usual decrease in tensile elongation associated with compounding, which is a disadvantage of PMMA, is ameliorated, with a higher tensile elongation than that of unmodified PMMA achieved. Therefore, we conclude that MPTMS-treated NFBCs are suitable nanofillers for PMMA reinforcement applications. In addition, this study revealed that the silane coupling agent used to treat the NFBC surface layer affects its interactions with the resin, in turn affecting the physical properties of the resulting composite and the optimal amount of NFBC required. Consequently, NFBCs are expected to be used as nanofillers for a variety of resins through the judicious choice of silane coupling agent.

## Figures and Tables

**Table 1:** Tensile mechanical properties of PMMA composites containing 0.05, 0.10, 0.25, 0.50, 1.0, and 2.0 wt% MPC 1, with those of PMMA (0 wt% MPC 1) provided as a reference (*n* = 5).

APC 1 Concentration/wt%	Tension Strain Limit/%	Tension Stress Limit/MPa	Young’s Modulus/GPa
0 ^1^	2.3 ± 0.3	22.1 ± 0.6	1.7 ± 0.1
0.05	2.7 ± 0.5	28.6 ± 0.7	2.4 ± 0.2
0.10	2.8 ± 0.4	34.2 ± 0.9	3.1 ± 0.2
0.25	2.4 ± 0.4	31.2 ± 1.1	2.9 ± 0.2
0.50	2.3 ± 0.4	24.0 ± 1.0	2.1 ± 0.2
1.0	2.2 ± 0.5	19.8 ± 0.6	1.6 ± 0.1
2.0	1.9 ± 0.7	16.2 ± 1.4	1.4 ± 0.2

^1^ PMMA reference.

## Data Availability

The data presented in this study supporting the results are available in the main text. Additional data are available upon reasonable request from the corresponding author.
